# Preparation of Structure-Function Integrated Layered CNT/Mg Composites

**DOI:** 10.3390/ma17102191

**Published:** 2024-05-07

**Authors:** Shiping Deng, Linchi Zou, Zengxiang Liao, Zhijie Lin

**Affiliations:** College of Materials Science and Engineering, Fujian University of Technology, 3 Xueyuan Road, University Town, Fuzhou 350118, China; 15655523862@163.com (S.D.); zx2221604027@126.com (Z.L.); zjlin@fjut.edu.cn (Z.L.)

**Keywords:** Mg-matrix composites, mechanical properties, damping properties, electromagnetic shielding effectiveness

## Abstract

Magnesium (Mg)-matrix composites have excellent damping and electromagnetic shielding properties. However, the mismatch between their strength and toughness limits their wide application. The aim of this work is to overcome the strength-toughness mismatch by constructing micro- and nanostructures while maintaining the good functional properties of Mg-matrix composites. Electrophoretic deposition (EPD) was used to spread carbon nanotubes (CNTs) out evenly on a Mg foil matrix. After spark plasma sintering (SPS), the grain organisation was refined, and the interlayer bonding was strengthened by hot rolling deformation. Finally, the microstructure, mechanical properties, damping properties, and electromagnetic shielding properties of the composites were analysed. Compared with the pure Mg laminates, the tensile strength and elongation of the CNT/Mg laminates were increased by 6.4% and 108.4%, respectively, with the significant improvement in toughness resulting from the increase in energy required for crack propagation due to the laminate structure. When the total rolling deflection reaches 80%, the interlayer bond strength of the material is significantly increased, the grain is further refined, and the strength and elongation of the composite material reaches the optimum, with the tensile strength reaching 241.70 MPa and the elongation reaching 6.90%. The interlayer interface and grain refinement also affected the damping Mg and electromagnetic shielding effect of the composites. This work provides an experimental idea for the preparation of high-performance structure-function integrated Mg-based materials.

## 1. Introduction

Mg, as the lightest structural metal, possesses high specific strength and stiffness, making it a promising material for lightweight applications. Additionally, Mg exhibits exceptional properties such as high damping, good vibration and noise damping, and excellent electromagnetic shielding [1,2]. To meet the demands of industrial applications, incorporating an exceptional reinforcing phase into the Mg matrix is an effective approach to obtain materials with excellent overall performance [3,4]. CNTs are commonly used as reinforcements in Mg-matrix composites due to their high strength, modulus, and low density. However, the plasticity of Mg is limited by its hexagonal close packed (HCP) structure, resulting in a strength and toughness mismatch even with nanoscale reinforcements, which significantly hampers the application of Mg-matrix composites [5,6].

The ‘brick-mud’ structure, exemplified by mother-of-pearl shells, exhibits exceptional toughness while maintaining high strength due to its unique structure [7]. Nanolaminates, resembling shell-pearl layers, have been employed in metal matrix composites to enhance strength without compromising toughness [8,9]. This nanolamellar structure offers a potential solution to balance the trade-off between strength and toughness in metal matrix composites. Inspired by these findings, the incorporation of a bionic lamellar structure into Mg-matrix composites presents a feasible method to achieve concurrent enhancement of material strength and toughness.

Although Mg-matrix composites reinforced with CNTs have shown success in improving strength and toughness [10,11,12,13], several challenges remain unresolved, and the preparation method and toughening mechanism are not fully developed. Due to the ultra-high strengthening efficiency of the CNTs/Mg layered structure, this structure is considered to be the main cause of high back stresses, but its toughening mechanism has not yet been investigated [10]. Flake powder metallurgy [14], EPD [10], and spray deposition [13,15] are commonly used methods to prepare carbon nanophase-reinforced layered metal-matrix composites. However, the flammable and oxidative properties of Mg powder render the flake powder metallurgical method unsuitable for preparing Mg-matrix composites.

Mg-matrix materials exhibit desirable functional properties, including damping and electromagnetic shielding capabilities. To date, numerous studies have been conducted on the damping properties of Mg-matrix composites, yet there remains considerable uncertainty regarding the damping capacity of Mg-matrix composites in response to temperature fluctuations. It was found that Mg-matrix composites are mainly affected by dislocation damping at room temperature and by a grain boundary damping mechanism and interface damping mechanism at high temperature, but the turning point is still unknown [16]. Furthermore, the impact of grain refinement on the damping characteristics of composites has not been fully elucidated [17]. It has been demonstrated that the incorporation of laminar structures enhances the electromagnetic shielding efficacy of composites [18]. The laminated structure prolongs the propagation path of electromagnetic waves inside the Mg-matrix composites, and the interlayer interface increases the multi-layer reflective capability, which enhances the electromagnetic shielding performance of Mg-matrix composites [19].

In this paper, CNTs were selected as the reinforcement and pure Mg foil as the matrix, and the layered CNTs/Mg-matrix composites were prepared by SPS using the EPD process. With the goal of achieving effective interlayer bonding, the interfacial bonding was strengthened, the interlayer spacing was controlled, and hot rolling was employed as the plastic deformation method. The interrelationship between the organization, mechanical properties, damping properties and electromagnetic shielding of layered CNTs/Mg-matrix composites was investigated. This study is expected to provide a new idea for the preparation of high-performance structural-functional integrated Mg-based materials.

## 2. Materials and Methods

**Figure 1:**
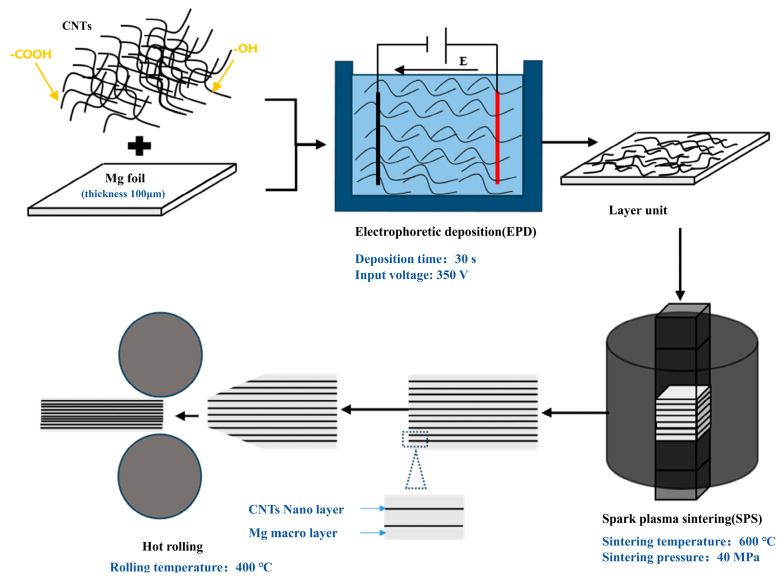
Preparation method.

Figure 1 delineates the preparation process of layered CNTs/Mg composites. Initially, CNTs/Mg layered matrix elements were acquired via EPD. Subsequently, these layered matrix elements were assembled and transformed into bulk composites through SPS. Ultimately, the sintered composites underwent deformation via hot rolling.

### 2.1. Preparation Process

#### 2.1.1. CNTs’ Pre-Dispersion Treatment

Multi-walled CNTs, featuring a diameter of 10–20 nm and a length of 5–15 µm, were provided by Shenzhen NanoPort Ltd. (Shenzhen, China). To improve the dispersibility of CNT in isopropanol, oxygen-containing functional groups were introduced on the surface of CNT by acidification (H_2_SO_4_:H_2_O_2_ = 7:3), followed by repeated dilution to pH = 7 and vacuum drying at 110 °C for 12 h. The dried CNTs and Al(NO_3_)_3_-9H_2_O were added into isopropanol solvent, and then ultrasonicated to form a stable and homogeneously dispersed suspension for EPD.

#### 2.1.2. Preparation of Layered Primitives

The EPD suspensions were prepared by ultrasonically dispersing 0.4 g/L of Al(NO_3_)_3_ and 0.2 g/L of CNTs in isopropanol for 6 h. The cathode of the EPD was a Mg foil (100 mm × 100 mm × 100 nm), while the anode was a stainless-steel sheet of the same dimensions, with a spacing of 6 cm between the Mg foil sheet and the stainless steel. The deposition of CNTs on the Mg foil was determined by the applied voltage and deposition time, which were set at 350 V and 30 s, respectively.

#### 2.1.3. Spark Plasma Sintering

The layered primitives, formed using the EPD method, were cut into 30 × 30 mm^2^ pieces and stacked alternately with one layer of Mg and one layer of CNTs, totalling approximately 46 pieces per sample. The stacked samples were wrapped in graphite paper and placed into graphite moulds of the appropriate size for SPS. During the sintering process, the pressure is gradually increased to 40 MPa as the temperature increases to a set maximum temperature. The temperature increased from room temperature to 500 °C within 10 min of the start of sintering, and then at 25 °C/min from 500 °C to 600 °C. The sintered composites were held at 600 °C for 9 min before cooling to room temperature.

#### 2.1.4. Hot Rolling

The composites in their sintered state were held at 400 °C for 15 min before undergoing deformation through rolling. The rolling process consisted of single passes at 20% and 40% deformation, followed by two passes at 40% and 33% deformation, resulting in a total deformation of 60%. To achieve 80% deformation, the composites were rolled at 40% and then at 33% for three passes. Prior to each rolling pass, the composites were held at 400 °C for 15 min.

### 2.2. Material Characterization

**Figure 2:**
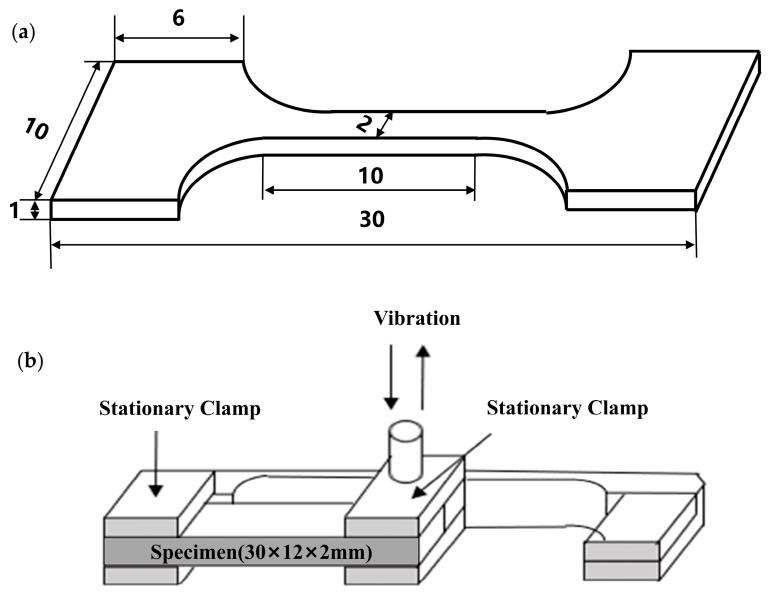
(**a**) Schematic diagram of tensile specimen dimensions; (**b**) schematic diagram of the single cantilever damping test setup.

Field emission scanning electron microscopy (FE-SEM, Nova Nano SEM 450, Hillsborough, OR, USA) was used to observe the morphology of CNTs deposited on the surface of Mg foils and the fracture morphology of tensile specimens. Transmission electron microscopy (TEM, JEM-2100F, Tokyo, Japan) was used to study the interlayer interface of the CNTs/Mg composites. To prepare the TEM samples, a 300 μm specimen was first cut along the thickness of the composite layer. A φ3mm metal disc was obtained by hand grinding to 50 μm and stamping. The thickness of the sample was further thinned to perforation using an ion thinning process. The tensile properties were tested using an electronic universal testing machine (Instron 2382). The tensile specimens of the composites were cut along the normal direction of the layer thickness. The strain rate for both pure Mg and the composites was set at 0.6 mm/min. Tensile testing was conducted using dog bone-shaped specimens with a scale length of 10 mm, a width of 2 mm, and a thickness of 1 mm, as shown in Figure 2a. Yield strength (YS) and ultimate tensile strength (UTS) were determined by stretching three specimens of each material. The damping behaviour of the composites was analysed at various frequencies ranging from room temperature to 350 °C using a Dynamic Thermomechanical Analyzer (DMA 242E Artemis) from NETZSCH, Selb, Germany. The damping experiments were conducted in a single cantilever mode, and the fixture structure is illustrated in Figure 2b. The test sample had dimensions of 30 mm × 12 mm × 2 mm. The electromagnetic shielding effectiveness of the Mg-based nanocomposites in the 8.2–12.4 GHz band was investigated using a vector network analyser (E5071C) from Keysight (Santa Rosa, CA, USA). The experiments were conducted using the waveguide method with test samples measuring 10.1 mm × 22.8 mm × 2 mm. The formula for calculating the electromagnetic shielding effectiveness is shown below.

(1) $$SE=-10lgT=-10lg{\left(S21\right)}^{2}$$

(2) $$SER=-10lg(1-R)=-10lg(1-{\left(S11\right)}^{2})$$

(3) $$SEA=-10lg\left[\left(T/\left(1-R\right.\right)\right]=-10lg\left[{\left(S21\right)}^{2}/\left(1-{\left(S11\right)}^{2}\right)\right]$$

## 3. Results

### 3.1. Distribution of CNTs on Mg Foil Surface

**Figure 3:**
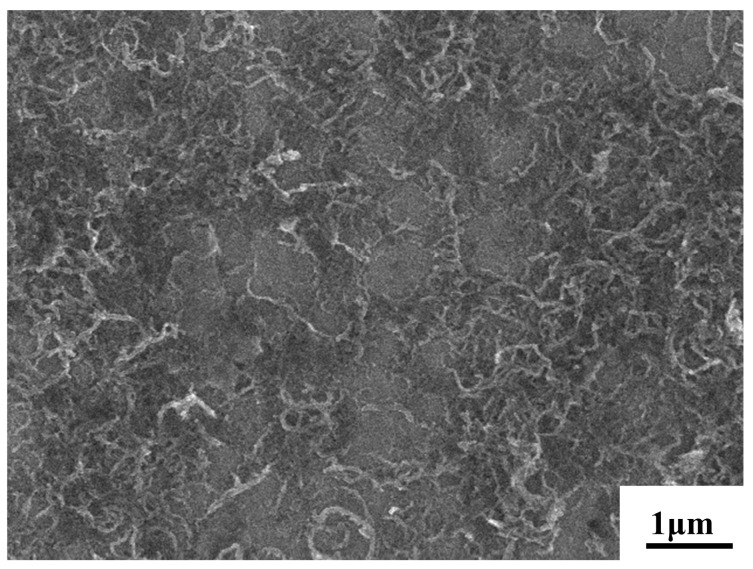
Scanning topography of CNTs on the surface of Mg foils.

Figure 3 displays the scanned morphology of CNTs deposited on the surface of Mg foil. The CNTs are visible as light-coloured long stripes on the surface of the Mg foil. Following EPD, the CNTs on the surface of the Mg foil were uniformly distributed without obvious agglomeration.

### 3.2. Mechanical Properties of Laminated CNTs/Mg Composites

**Figure 4:**
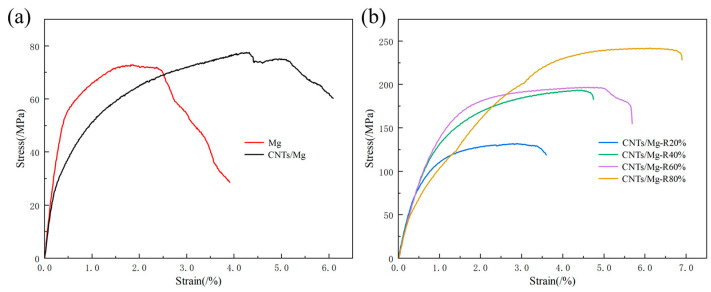
(**a**) Stress-strain curves of laminated pure Mg as well as sintered state laminated CNTs/Mg composite specimens; (**b**) stress-strain curves of rolled state laminated CNTs/Mg composite specimens.

Figure 4a displays stress-strain curves for the mechanical properties of laminated pure Mg contrast matrix and laminated CNTs/Mg composite specimens. Table 1 details the values of tensile properties of laminated pure Mg and composites at different rolling deflections. Under the same sintering conditions, the tensile strength and elongation of the layered CNTs/Mg increased by 6.4% and 108.4%, respectively, compared to the layered pure Mg specimens. Compared with pure Mg, the laminated CNTs/Mg composites improved the tensile strength and toughness significantly. This indicates that the laminate structure well-balances the conflict between strength and toughness of Mg-matrix composites, and that CNTs play an effective role as reinforcement in improving the performance of composites. Figure 4b shows the stress-strain curves for the mechanical properties of laminated CNTs/Mg composite specimens after hot rolling. Following 40% deformation in a single rolling, the tensile strength of the sintered composites reached 193.47 MPa, representing a 149.5% increase in comparison to the tensile strength of the sintered specimens. The composites with 40% deformation in a single pass were then rolled, and the total deformation of the composites was further increased, while the strength and elongation were also increased by increasing the number of rolling passes. The increase in rolling deformation results in a reduction in the grain size of the composites and the interlayer bond strength increases with the increase in deformation. When the total deformation of rolling reaches 80%, the strength and toughness of the composite material reach the maximum; its tensile strength reaches 241.70 MPa and elongation reaches 6.90%. The strength of the composite material after rolling with large deformation has been significantly improved, and the toughness has also been improved. This shows that the rolling process has a more significant effect in strengthening the interlayer bonding of the material to improve the strength of the material. In summary, compared with other Mg-based composites [20,21,22,23,24,25,26,27], CNT/Mg laminated composites show excellent toughening effect while improving strength.

#### 3.2.1. Strengthening Mechanism of Laminated CNTs/Mg Composites

**Figure 5:**
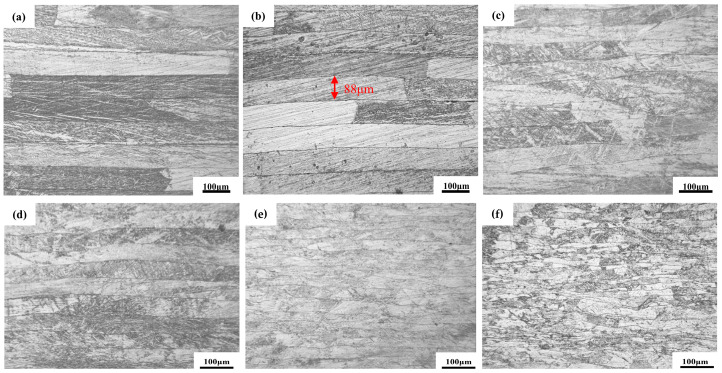
Metallographic histograms of laminated CNTs/Mg composites: (**a**) laminated pure Mg; (**b**) laminated CNTs/Mg composites; (**c**) 20% rolled deformation; (**d**) 40% rolled deformation; (**e**) 60% rolled deformation; (**f**) 80% rolled deformation.

The optical microscopic observation of laminated pure Mg and laminated CNTs/Mg composites provides insight into the strengthening mechanism, as shown in Figure 5. After corrosive agent corrosion, both the laminated pure Mg and sintered state composites appeared as neatly laminated rows on the thickness surface, with an average interlayer spacing of 90 µm, slightly smaller than the original Mg foil thickness. The growth of grains in the direction perpendicular to the interlayer was restricted by the interlayer, resulting in the grains being predominantly rectangular and brick-shaped with overlapping grain boundaries at the interface, as shown in Figure 5a,b. Following hot rolling, the original coarse grains gradually disappeared, and the grains were further refined with increased deformation, leading to improved mechanical properties of the composite material. This phenomenon was also confirmed by observing the RD surface through optical microscope as shown in Figure 5c–f. In the rolled state, the composites maintained an interlayer interface, and as the total deformation increased, the average thickness of the interlayer decreased, while the interface underwent twisting deformation. After a single rolling pass with large deformation, no significant interlaminar cracking was observed. This can be attributed to the bridging effect of the large deformation force between the interlaminar layers. Subsequent to 80% of the total deformation from rolling, a rolled shear band was observed in the optical microstructure at an angle of approximately 35° to the rolling direction, as shown in Figure 5f. Further increasing the number of passes to enhance the total deformation may result in shear failure. In summary, the process of rolling deformation serves to refine the matrix organization, control the interlayer distance, strengthen the interlayer bonding, and effectively improve the strength of the composite material.

**Figure 6:**
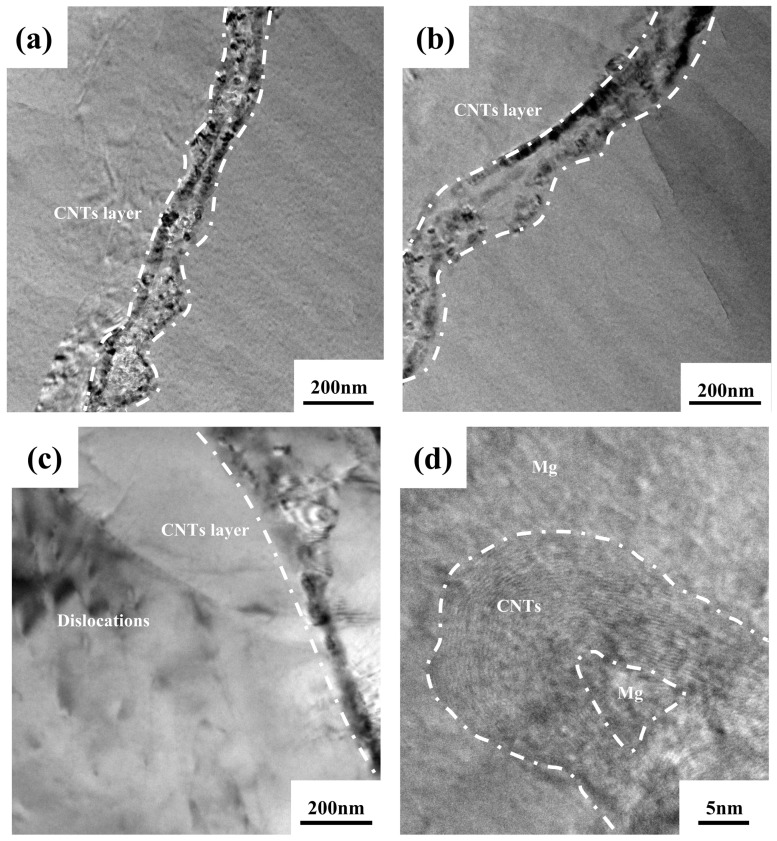
TEM image of CNTs/Mg composite material: (**a**) CNTs are linearly distributed, some of which are embedded in the matrix; (**b**) dislocations accumulate at grain boundaries; (**c**) dislocations are clustered around the CNTs; (**d**) CNTs are mechanically connected to the Mg matrix.

To investigate the interfacial bonding between CNTs and the Mg matrix in the CNTs/Mg composites, TEM was used to analyse the samples. The TEM images of the CNTs/Mg composites for a sample with a diameter of 3 mm are shown in Figure 6. Figure 6a shows clear interlayer structural features between adjacent Mg layers, with CNTs visible both intertwined and embedded in the Mg matrix. CNTs in the interlayers are able to transfer the load and thus improve the mechanical properties of the composites. Due to the presence of CNTs between the Mg layers, the grains are restricted from growing beyond the interlayer interface, thus effectively suppressing grain oversize, as shown in Figure 6b. Due to the different coefficients of thermal expansion between the CNTs and the Mg matrix, the thermal mismatch between the reinforcement and the Mg matrix during the preparation process enhances the dislocation density around the reinforcement. As shown in Figure 6c, a large number of dislocations are stacked at the interface position between CNTs and Mg matrix. In order to further observe the bonding of CNTs with Mg matrix, high-resolution observation of Figure 6b was performed, and it can be seen that the CNTs are tightly mechanically bonded to the Mg matrix at the interlayer interface, as shown in Figure 6d.

#### 3.2.2. Toughening Mechanism of Laminated CNTs/Mg Composites

**Figure 7:**
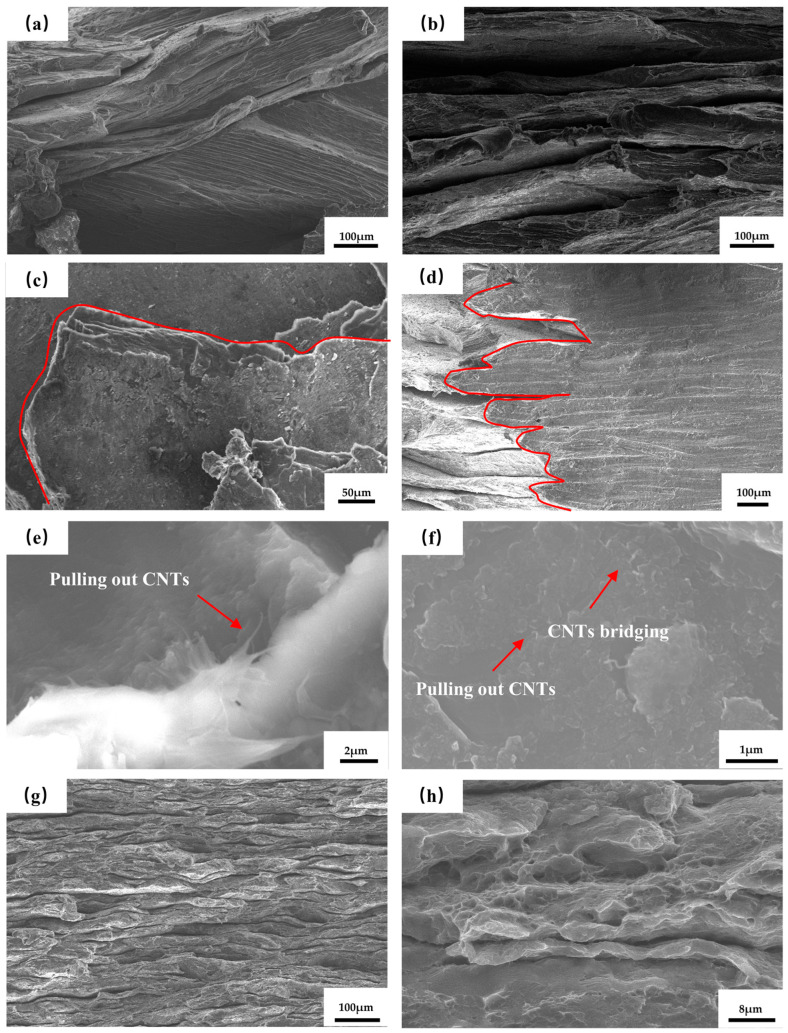
Tensile fracture sweep of CNTs/Mg composites: (**a**) Scan of tensile fracture of laminated Mg; (**b**) scan of tensile fracture of laminated CNT/Mg composite; (**c**) cracks deflected in the interlayer; (**d**) lateral macroscopic morphology of laminated CNT/Mg composites; (**e**) CNTs pulling out at the fracture; (**f**) CNTs pulling out and bridging; (**g**) low magnification image of 80% deformation composite fracture scan; (**h**) high magnification image of 80% deformation composite fracture scan.

In order to further observe the fracture characteristics of the materials and analyse the fracture mechanism, the fracture of laminated pure Mg and laminated CNTs/Mg composites were observed, and the fracture morphology is shown in Figure 7. The tensile fracture of laminated pure Mg does not exhibit obvious delamination, with adjacent Mg layers tightly bonded together, as shown in Figure 7a. Compared to the laminated pure Mg, the sintered CNTs/Mg composite material shows a more complete laminated structure and smooth interfaces. The fracture surface of the composite material is irregular and serrated, contrasting with the flat fracture surface of pure Mg, as shown in Figure 7b,c. Observations at the interlayer interface of the fracture at high magnification, as shown in Figure 7e,f, show that the CNTs pull out from the fracture as well as the bridging phenomenon, which proves that the CNTs can effectively transfer the loads. The fracture morphology reveals a sawtooth-like and elongated crack propagation path at the layer interfaces, as shown in Figure 7c. The macroscopic lateral morphology of the tensile fracture shows that the crack propagation is stepwise, as shown in Figure 7d. This is due to the inhibiting effect of the layer interfaces on crack propagation. When the main crack reaches the layer interface region, it deflects, resulting in lateral extension within the layers before propagating longitudinally [28,29,30,31]. These conditions increase the energy consumption during crack propagation and enhance the fracture toughness of the material.

To analyse the fracture behaviour and mechanisms of the rolled composite material, we scanned and observed the tensile fracture of the composite material, as shown in Figure 7g,h. The tensile fracture of the composite material in the rolled state exhibits a more compact laminated structure compared to the sintered state. Additionally, there is no delamination between the layers, indicating that rolling promotes bonding between the interfaces. With the decrease in layer thickness, the number of layer interfaces increases. The increased number of layer interfaces implies an increased resistance to crack propagation. Figure 7g shows that after undergoing significant plastic deformation, some layers become more curved. Upon high-magnification observation of the fracture surface, tear bands and toughening dimples are observed on the fracture surface of the composite material when subjected to 80% rolling deformation, as shown in Figure 7h.

### 3.3. Damping Characteristics of Laminated CNTs/Mg Composites

**Figure 8:**
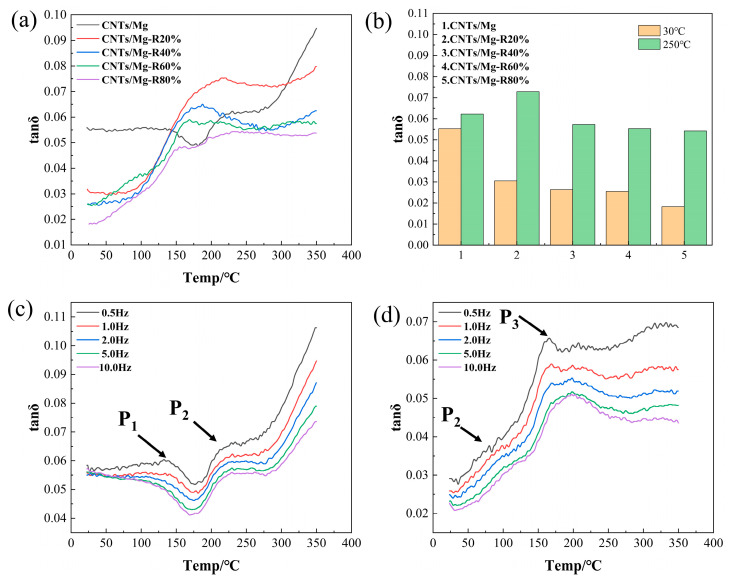
Effect of rolling deflection on damping characteristics: (**a**) Damping-test temperature curves (tanδ~T) of laminated CNTs/Mg composites with different deformations at 1 Hz vibration frequency; (**b**) damping performance of laminated CNTs/Mg composites with different deformations at 30 °C and 250 °C (1 Hz); (**c**) damping-test temperature curves (tanδ~T) of sintered composites at different frequencies; (**d**) damping-test temperature curves (tanδ~T) of the composites with 60% of total deformation at different frequencies.

Figure 8a shows the temperature curves (tanδ~T) of CNTs/Mg composites with varying deformations at a vibration frequency of 1 Hz. The damping values of the sintered composites remain relatively stable between room temperature and 140 °C, with a gradual decrease starting at 140 °C and a gradual increase starting at 180 °C, forming a peak at approximately 230 °C. The damping value then continues to increase gradually at higher test temperatures (270–350 °C). In comparison to the sintered composites, the rolled composites exhibit a lower damping value at room temperature. The damping value of the material increases gradually with temperature from room temperature to −120 °C, and then increases rapidly beyond 120 °C. Within a certain temperature range, the damping value is higher than that of the sintered state composites. An internal dissipation peak is observed between 160 °C and 220 °C. As the test temperature increases, the damping values of the composites remain relatively stable.

Figure 8b illustrates the damping performance of CNTs/Mg composites at room temperature (30 °C) and high temperature (250 °C) under different deformations. At room temperature, the damping value of the composites gradually decreases with increasing deformation. The damping value of the composites with 80% deformation decreases by 67.04% compared to that of the sintered state specimens. At high temperatures, the damping value of composites with 20% deformation increased by 17.11% compared to the sintered specimen. However, at higher deformations, the damping value decreased with increasing deformation. Specifically, the damping value of composites with 80% deformation decreased by 13.85% compared to the sintered specimen.

In the low-temperature interval, the composites are mainly affected by dislocation damping. The CNTs act as pinning agents for dislocations within the composites, causing them to wind and anchor at the points of contact with the Mg matrix. This process effectively reduces both the length and quantity of mobile dislocations. When subjected to applied stress, internal dissipation arises solely from dislocations engaged in string resonance motion (P_1_). Following deformation of the composite material, the matrix grain undergoes refinement, thereby enhancing the strength of the composite material. However, this refinement also results in an increase in grain boundaries, which can impede dislocation motion and consequently lead to a decline in damping performance [32]. At elevated temperatures, the damping behaviour of the composites is primarily influenced by grain boundary damping and interface damping. With increased temperatures, grain and phase boundaries become more prone to sliding, and grain boundary slip, which is challenging to initiate at room temperature, becomes feasible [18,33]. The sintered composites produce an internal depletion peak (P_2_) near 220 °C, and the P_2_ peak is characterized by thermal activation, which is presumed to be a grain boundary slip peak. Subsequent significant deformation of the grains causes them to transition into a non-equilibrium state, resulting in a notable reduction in the activation energy required for grain boundary slip. Consequently, the P_2_ peak of the rolled state composites shifts towards the low-temperature region [34]. As the temperature rises further, the rolled state composites exhibit an internal consumption peak (P_3_) spanning the range between 160 °C and 230 °C. The peak temperature of the P_3_ peak remains nearly independent of frequency. Nonetheless, with increasing deformation, the peak temperature of the P_3_ peak shifts towards the lower temperature region, indicative of a recrystallization peak [35,36].

### 3.4. Electromagnetic Shielding Performance of Laminated CNTs/Mg Composites

**Figure 9:**
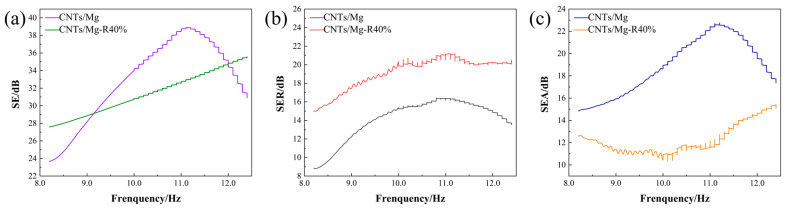
Shielding effectiveness of composites before and after rolling with 40% deformation: (**a**) Shielding effectiveness; (**b**) reflection effectiveness; (**c**) absorption effectiveness.

Figure 9 displays the electromagnetic shielding effectiveness curves of sintered composites with a thickness of 2 mm and composites after 40% rolling deformation in the 8.2–12.4 GHz band. The material shielding effectiveness is obtained by adding the absorbing and reflecting effectiveness. The average electromagnetic shielding effectiveness values of the composites in this band are presented in Table 2. The average electromagnetic shielding effectiveness value of the composites in this band after rolling is 31.3 dB, with an average reflective property value of 19.1 dB and an average absorptive effectiveness value of 12.2 dB. The electromagnetic shielding effect of the composites after 40% rolling was reduced by 5.2% in the average value of this band, the reflecting effect was improved by 35.5% compared to the average value of the sintered specimens, and the average value of the wave-absorbing properties was reduced by 35.4%.

The analysis shows that the sintered state composites are refined and the grain size is reduced after the composites are rolled. This grain refinement increases the area of grain boundaries, which act as impedance discontinuous interfaces, thereby increasing the reflection of electromagnetic waves and the reflective efficiency of the metal. The rolling process results in an increase in the number of interlayer interfaces, which leads to enhanced reflection and multiple reflection losses of incident electromagnetic waves [37]. After the composite is rolled, its base texture becomes stronger and there are more grains of the same orientation. The conductive network formed between the grains is connected to each other in a larger plane, and this continuity of the conductive network increases the reflection loss of the metal to electromagnetic waves [38]. The absorption loss of the material is determined by the thickness of the shielding layer, as well as its magnetic and electrical conductivity. Higher conductivity results in higher absorption loss. The theory of metal conductivity suggests that grain boundaries, as crystal defects, act as electron scattering centres. An increase in grain boundary density shortens the mean free range of electrons, reducing the metal’s electrical conductivity and affecting its electromagnetic shielding performance [39].

## 4. Conclusions

Compared to pure Mg with a layered structure, the tensile strength and elongation of CNTs/Mg composites with a layered structure increased by 6.4% and 55.5%, respectively. The presence of interlayer interfaces impedes crack propagation and restricts interlayer grain growth. Additionally, the CNTs are firmly bonded to the matrix, effectively transferring load. These results confirm the strengthening and toughening effects of CNTs in laminated structures. After undergoing rolling deformation, the grains of the composites were refined, interlayer bonding was strengthened, and the overall strength increased with increasing deformation. The highest strength and toughness of the composite material were achieved when the total rolling deformation reached 80%, resulting in a tensile strength of 241.70 MPa and elongation of 6.90%.

The damping properties of the composites gradually decrease at room temperature as the rolling deformation increases. At 30 °C, the damping value of the composite with 80% deformation decreased by 67.04% compared to the sintered-state specimen. However, at a high temperature of 250 °C, the damping value of the composites with 20% deformation increased by 17.11% compared to the sintered specimens. Nevertheless, at higher deformations, the damping value decreased with further increases in deformation. The damping-test temperature curves of composites with different deformations exhibited three internal depletion peaks. The P_1_ peak was mainly attributed to the string resonance motion of dislocations. The P_2_ peak was characterised by thermal activation induced by grain boundary slip. The P_3_ peak only appeared in the rolled composites, and its height decreased with increasing frequency, suggesting it to be a recrystallization peak.

In the 8.2–12.4 GHz band, the electromagnetic shielding effectiveness of the laminated CNTs/Mg composites decreased by 5.2% after 40% rolling. The reflection effectiveness increased by 35.5% compared to the sintered-state specimen. However, the wave-absorbing property decreased by 35.4%.

## Figures and Tables

**Table 1:** Mechanical properties of laminated pure Mg and laminated CNTs/Mg composites.

Materials	YS (MPa)	UTS (MPa)	εf (%)
Mg	56.92 ± 1.21	72.88 ± 2.51	2.48 ± 0.18
CNTs/Mg	34.21 ± 1.78	77.52 ± 2.17	5.17 ± 0.62
CNTs/Mg (R20%)	92.34 ± 4.49	131.89 ± 6.42	3.60 ± 0.54
CNTs/Mg (R40%)	114.28 ± 3.15	193.47 ± 2.09	4.75 ± 0.35
CNTs/Mg (R60%)	123.81 ± 4.43	196.77 ± 3.13	5.68 ± 0.11
CNTs/Mg (R80%)	180.98 ± 8.63	241.70 ± 10.16	6.90 ± 0.51

**Table 2:** Average values of shielding effectiveness of composites before and after rolling at 40% deformation in the 8.2–12.4 GHz band.

Material	Average Shielding Effectiveness (dB)	Average Reflection Effectiveness (dB)	Average Absorption Effectiveness (dB)
CNTs/Mg	33.0	14.1	18.9
CNTs/Mg-R40%	31.3	19.1	12.2

## Data Availability

Data are contained within the article.
